# Supplementary material for: Detailed molecular and epigenetic characterization of the pig IPEC-J2 and chicken SL-29 cell lines
Source: iScience. 2023 Feb 20;26(3):106252. doi: 10.1016/j.isci.2023.106252 (PMC10018572; doi:10.1016/j.isci.2023.106252)
Supplement: Data S1. Complete homer output for identified motifs in Pig IPECJ-2, related to Table 2 — Homer motif analysis results for histone modifications H3K4me1, H3K4me3, H3K27ac, and enhancer elements of pig IPECJ2 cell line. P-values >1e-10 are possible false positives. Within each folder (e.g. peak_files_CTCF) are the html files showing the identified motifs when using homer (e.g. homerResults.html). [file mmc2.zip › S5/Pig_IPECJ_2/peak_files_enhancer/homerResults.html]

peak\_files\_enhancer/ - Homer de novo Motif Results


# Homer *de novo* Motif Results (peak\_files\_enhancer/)

Known Motif Enrichment Results  
Gene Ontology Enrichment Results  
If Homer is having trouble matching a motif to a known motif, try copy/pasting the matrix file into
STAMP  
More information on motif finding results: HOMER
| Description of Results
| Tips
  
Total target sequences = 36644  
Total background sequences = 36590  
\* - possible false positive  

|  |  |  |  |  |  |  |  |  |
| --- | --- | --- | --- | --- | --- | --- | --- | --- |
| Rank | Motif | P-value | log P-pvalue | % of Targets | % of Background | STD(Bg STD) | Best Match/Details | Motif File |
| 1 | A G C T C A T G C G T A A T C G C G A T G T A C T C G A A G C T A G T C A T C G | 1e-469 | -1.080e+03 | 11.80% | 5.49% | 82.9bp (90.8bp) | Fra1(bZIP)/BT549-Fra1-ChIP-Seq(GSE46166)/Homer(0.980) More Information \| Similar Motifs Found | motif file (matrix) |
| 2 | A C G T G T A C C T G A T G A C G T C A C A G T C G A T G A T C G A T C C G A T | 1e-177 | -4.098e+02 | 19.34% | 13.94% | 83.4bp (86.7bp) | TEAD3/MA0808.1/Jaspar(0.962) More Information \| Similar Motifs Found | motif file (matrix) |
| 3 | C T G A A G C T A G C T G C A T T G A C G T A C C A G T T C G A | 1e-80 | -1.845e+02 | 38.55% | 33.80% | 84.9bp (90.2bp) | EWS:ERG-fusion(ETS)/CADO\_ES1-EWS:ERG-ChIP-Seq(SRA014231)/Homer(0.903) More Information \| Similar Motifs Found | motif file (matrix) |
| 4 | A C T G A C G T A C G T A T G C C G A T A T C G C A G T G T C A C T G A C T G A G A T C G T A C C G T A | 1e-64 | -1.494e+02 | 0.14% | 0.00% | 84.3bp (25.8bp) | FOXN3/MA1489.1/Jaspar(0.717) More Information \| Similar Motifs Found | motif file (matrix) |
| 5 | G C A T A C T G A C G T A C G T A C G T C G T A C G T A T C G A G T C A G T C A | 1e-61 | -1.417e+02 | 46.07% | 41.77% | 84.9bp (87.4bp) | CHR(?)/Hela-CellCycle-Expression/Homer(0.754) More Information \| Similar Motifs Found | motif file (matrix) |
| 6 | G A C T C A G T G T C A A G T C C G A T A C T G G A T C A T G C C A G T C A T G | 1e-56 | -1.306e+02 | 8.78% | 6.62% | 83.9bp (91.5bp) | ETV1/MA0761.2/Jaspar(0.739) More Information \| Similar Motifs Found | motif file (matrix) |
| 7 | A T C G A C G T A C T G A C G T T G C A C G T A A C G T C A G T A C G T G C A T G T A C C G T A C T A G | 1e-54 | -1.262e+02 | 0.12% | 0.00% | 88.3bp (0.0bp) | PB0146.1\_Mafk\_2/Jaspar(0.616) More Information \| Similar Motifs Found | motif file (matrix) |
| 8 | T G C A G C T A T G A C G C T A C A T G A T C G T A G C G A T C A G C T G C A T | 1e-54 | -1.256e+02 | 34.62% | 30.81% | 84.2bp (90.0bp) | ZFX(Zf)/mES-Zfx-ChIP-Seq(GSE11431)/Homer(0.764) More Information \| Similar Motifs Found | motif file (matrix) |
| 9 | C T A G C A G T C G A T C T G A C A G T A T C G C G A T T C A G G T C A G T A C | 1e-53 | -1.241e+02 | 25.82% | 22.38% | 83.7bp (87.3bp) | USF1/MA0093.3/Jaspar(0.805) More Information \| Similar Motifs Found | motif file (matrix) |
| 10 | C G T A A G T C C T G A A T G C T A G C A T G C C G A T C T A G | 1e-43 | -9.998e+01 | 47.19% | 43.59% | 84.8bp (88.3bp) | TBX3/MA1566.1/Jaspar(0.758) More Information \| Similar Motifs Found | motif file (matrix) |
| 11 | C A T G T A G C A G C T T A C G G C A T C A G T A G C T A T C G | 1e-40 | -9.416e+01 | 15.50% | 13.07% | 84.5bp (89.2bp) | PRDM4/MA1647.1/Jaspar(0.811) More Information \| Similar Motifs Found | motif file (matrix) |
| 12 | A C G T T G A C G C A T C T A G T C A G A T C G T G A C T G C A C T A G C A T G | 1e-40 | -9.212e+01 | 26.04% | 23.07% | 84.2bp (90.2bp) | ZNF416(Zf)/HEK293-ZNF416.GFP-ChIP-Seq(GSE58341)/Homer(0.806) More Information \| Similar Motifs Found | motif file (matrix) |
| 13 | G T C A G C T A G T C A A G C T T A C G C G A T G A C T C G A T T C G A A G T C | 1e-39 | -9.118e+01 | 13.34% | 11.11% | 84.8bp (86.1bp) | FOXA1/MA0148.4/Jaspar(0.860) More Information \| Similar Motifs Found | motif file (matrix) |
| 14 | G A T C G T C A C T G A C T G A A C T G A G T C C G T A A T G C | 1e-39 | -9.027e+01 | 20.32% | 17.64% | 85.1bp (89.0bp) | SOX10/MA0442.2/Jaspar(0.704) More Information \| Similar Motifs Found | motif file (matrix) |
| 15 | A T C G T G A C C G A T C G T A C T G A T G A C G C A T A G T C | 1e-38 | -8.765e+01 | 20.48% | 17.84% | 85.2bp (89.9bp) | MafA(bZIP)/Islet-MafA-ChIP-Seq(GSE30298)/Homer(0.723) More Information \| Similar Motifs Found | motif file (matrix) |
| 16 | C T A G A T G C A G T C G C T A C G T A A C T G A G C T G A C T | 1e-35 | -8.086e+01 | 12.30% | 10.27% | 84.1bp (90.3bp) | SD0002.1\_at\_AC\_acceptor/Jaspar(0.782) More Information \| Similar Motifs Found | motif file (matrix) |
| 17 | C T A G C T A G A C T G T C A G C A G T C T G A T C G A C G T A C A T G C T A G C G A T T C G A C T A G | 1e-31 | -7.267e+01 | 0.10% | 0.01% | 84.4bp (0.0bp) | PPARE(NR),DR1/3T3L1-Pparg-ChIP-Seq(GSE13511)/Homer(0.652) More Information \| Similar Motifs Found | motif file (matrix) |
| 18 | A T G C C G T A G C T A G A C T G C A T C G A T A C T G A G C T A T G C A G T C G C A T A C T G T G A C | 1e-31 | -7.267e+01 | 0.10% | 0.01% | 82.1bp (28.5bp) | ZNF675(Zf)/HEK293-ZNF675.GFP-ChIP-Seq(GSE58341)/Homer(0.654) More Information \| Similar Motifs Found | motif file (matrix) |
| 19 | C T G A T A C G C A G T C T A G G T C A C T G A A C G T A G T C C G A T A C T G A C T G T C A G C G A T | 1e-30 | -6.925e+01 | 0.11% | 0.01% | 80.4bp (39.2bp) | DUX(Homeobox)/C2C12-Dux-ChIP-Seq(GSE87279)/Homer(0.624) More Information \| Similar Motifs Found | motif file (matrix) |
| 20 | C G T A A C G T C G T A A G T C A C G T A C G T A G T C C G T A A C T G A C T G C G T A A C T G C G T A | 1e-27 | -6.408e+01 | 0.09% | 0.01% | 73.7bp (52.6bp) | ELK1/MA0028.2/Jaspar(0.608) More Information \| Similar Motifs Found | motif file (matrix) |
| 21 | A G C T A G T C G C T A T A G C C G T A C A T G T A C G G A C T A G C T C G T A T C A G G T C A C G T A | 1e-27 | -6.408e+01 | 0.09% | 0.01% | 78.8bp (62.7bp) | GRHL2/MA1105.2/Jaspar(0.619) More Information \| Similar Motifs Found | motif file (matrix) |
| 22 | A C G T A C T G C G T A A G T C A T G C A C G T A G T C C G T A | 1e-24 | -5.743e+01 | 2.42% | 1.68% | 84.2bp (91.5bp) | THRb(NR)/Liver-NR1A2-ChIP-Seq(GSE52613)/Homer(0.911) More Information \| Similar Motifs Found | motif file (matrix) |
| 23 | A G C T C G T A A T C G G T C A A C G T G C T A T C G A T C A G | 1e-24 | -5.743e+01 | 17.81% | 15.79% | 85.1bp (87.8bp) | GATA2/MA0036.3/Jaspar(0.910) More Information \| Similar Motifs Found | motif file (matrix) |
| 24 | T C A G G T C A C G T A C T A G G A T C G A C T C T G A G T A C G C A T C G A T T G C A C G T A G T A C | 1e-21 | -4.927e+01 | 0.09% | 0.01% | 80.5bp (11.3bp) | PH0115.1\_Nkx2-6/Jaspar(0.718) More Information \| Similar Motifs Found | motif file (matrix) |
| 25 | C G T A A G T C A T G C A C G T A G T C A C G T G T C A C G T A | 1e-21 | -4.911e+01 | 5.63% | 4.55% | 85.9bp (90.4bp) | PB0049.1\_Nr2f2\_1/Jaspar(0.668) More Information \| Similar Motifs Found | motif file (matrix) |
| 26 | C G T A A G T C A T G C C G T A A G T C A C G T A G T C A C G T C G T A C G T A | 1e-20 | -4.637e+01 | 0.11% | 0.02% | 82.3bp (27.2bp) | NKX2-2/MA1645.1/Jaspar(0.752) More Information \| Similar Motifs Found | motif file (matrix) |
| 27 | T A C G T A G C A T G C T A G C T C A G A T C G T A G C A G T C A T C G A T C G | 1e-19 | -4.582e+01 | 6.19% | 5.10% | 82.8bp (86.4bp) | Zfx/MA0146.2/Jaspar(0.671) More Information \| Similar Motifs Found | motif file (matrix) |
| 28 | A C G T G T A C G T A C T A G C G T A C T C A G G T C A T C A G | 1e-19 | -4.578e+01 | 8.10% | 6.84% | 86.5bp (87.4bp) | Znf281/MA1630.1/Jaspar(0.712) More Information \| Similar Motifs Found | motif file (matrix) |
| 29 | C G T A T G A C G T C A A C G T C T A G A G C T C A T G C A G T A C T G C G A T C G A T C T G A T C A G | 1e-19 | -4.464e+01 | 0.12% | 0.02% | 90.9bp (61.7bp) | MXI1/MA1108.2/Jaspar(0.683) More Information \| Similar Motifs Found | motif file (matrix) |
| 30 | T G A C C A T G A C T G A T C G T C G A C A T G A T C G G T A C C T A G C T A G T C A G T C G A A G C T | 1e-19 | -4.456e+01 | 0.08% | 0.01% | 76.7bp (55.2bp) | ZNF148/MA1653.1/Jaspar(0.722) More Information \| Similar Motifs Found | motif file (matrix) |
| 31 | C G T A A G T C A C G T A G T C C G T A A C T G C G T A C G T A A G T C A C T G C G T A A G T C A C G T | 1e-18 | -4.314e+01 | 0.09% | 0.01% | 69.0bp (59.9bp) | Bach1::Mafk/MA0591.1/Jaspar(0.563) More Information \| Similar Motifs Found | motif file (matrix) |
| 32 | C G T A A C G T C G T A A G T C A C G T C G T A G T C A A C T G A G T C A C G T | 1e-18 | -4.256e+01 | 0.07% | 0.01% | 87.6bp (10.4bp) | GATA1/MA0035.4/Jaspar(0.652) More Information \| Similar Motifs Found | motif file (matrix) |
| 33 | T A G C T A C G T A G C A T C G T A G C T A C G T A C G T A G C A T C G T A G C A T C G T A C G T A C G | 1e-15 | -3.538e+01 | 1.55% | 1.09% | 80.9bp (83.9bp) | PB0199.1\_Zfp161\_2/Jaspar(0.678) More Information \| Similar Motifs Found | motif file (matrix) |
| 34 | A T G C A T C G A T C G A C T G T A G C C A T G A T G C A C T G | 1e-14 | -3.341e+01 | 3.25% | 2.58% | 85.1bp (81.8bp) | POL006.1\_BREu/Jaspar(0.714) More Information \| Similar Motifs Found | motif file (matrix) |
| 35 | A C G T C G T A C G T A A C T G C G T A C G T A A C T G A G T C A G T C A G T C | 1e-14 | -3.299e+01 | 0.08% | 0.01% | 75.6bp (71.0bp) | POL008.1\_DCE\_S\_I/Jaspar(0.658) More Information \| Similar Motifs Found | motif file (matrix) |
| 36 | C T A G C A T G C T A G C T A G T C A G A C T G G T A C T G A C T G C A T C G A T A G C A G T C G A T C | 1e-13 | -3.116e+01 | 0.08% | 0.01% | 76.6bp (38.0bp) | PLAGL2/MA1548.1/Jaspar(0.689) More Information \| Similar Motifs Found | motif file (matrix) |
| 37 \* | A G T C A G C T A C T G C G T A A C G T A G T C A C G T A C T G A G T C A G T C | 1e-11 | -2.673e+01 | 0.10% | 0.03% | 84.9bp (75.9bp) | GATA1/MA0035.4/Jaspar(0.664) More Information \| Similar Motifs Found | motif file (matrix) |
| 38 \* | T A G C A T G C G A T C A C T G G A T C G A T C T C A G G A T C G T A C A C T G G A T C T G A C T A G C | 1e0 | -2.050e+00 | 0.26% | 0.23% | 89.0bp (60.9bp) | PB0010.1\_Egr1\_1/Jaspar(0.656) More Information \| Similar Motifs Found | motif file (matrix) |
